# Development and validation of a quality assessment tool to assess online nutrition information

**DOI:** 10.1177/20552076231187249

**Published:** 2023-07-16

**Authors:** Cassandra H Ellis, J Bernadette Moore, Peter Ho, Charlotte EL Evans

**Affiliations:** 1 School of Food Science and Nutrition, 4468 University of Leeds, Leeds, UK; 2 The Nutrition Society, London, UK

**Keywords:** Nutrition, quality assessment, quality assessment tool, digital health, online information, validation

## Abstract

**Setting:**

The internet is an important source of health information but is unregulated. Little research has focused on the assessment of digital information related to nutrition.

**Aim:**

To develop and validate a novel online quality assessment tool (OQAT) for quality assessment of online nutrition information.

**Method:**

The OQAT was developed and validated in six distinct stages. After reviewing the literature, a framework and criteria were developed and formalised. Next, the quality assessment criteria were piloted on a subset of data and criteria refined. The established criteria were then validated against a previously validated assessment tool, and reliability was tested. Finally, the validated OQAT was used to assess the quality of articles from a 24-h collection period, 19 April 2021.

**Results:**

The final OQAT consisted of 10 key questions. Twenty-six news articles were assessed independently by two raters. Comparison of scores found moderate internal consistency (*α* = 0.382). Cohen's Kappa coefficient demonstrated high interrater agreement (*k* = 0.653, *p* < 0.001). The OQAT was tested on 291 relevant Uniform Resource Locators (URLs), which were determined to be either poor 3% (*n* = 9), satisfactory 49% (*n* = 144), or high-quality 48% (*n* = 139) articles. There was a statistically significant difference in OQAT scores between blogs, news articles, and press releases, *χ*^2^(2) = 23.22, *p* < 0.001, with a mean rank OQAT score of 138.2 for blogs, 216.6 for news articles, and 188.7 for press releases.

**Conclusion:**

This novel tool provides a reliable and objective method for assessing the quality of nutrition content online. It could potentially be used by researchers to assess the quality of online information in different settings and by organisations to inform readers of the quality of information being accessed.

## Introduction

The internet, including social media, is one of the most important sources of nutrition information for the general population.^1^ However, it is largely unregulated.^2^ The quality of online nutrition information is important as misinformation leads consumers to believe that the scientific evidence is contradictory^3^ and negatively influences consumer beliefs.^4^ Additionally, contradictory information increases uncertainty about health research more generally. Furthermore, the perception of conflicting health reports in the media can increase negative attitudes towards health policy and reduce adherence to guidelines.^5^ Nutrition guidelines are a key component of the wider health agenda with the World Health Organization making recommendations on diet quality aimed at individuals and the wider food environment. However, there is a rise in self-proclaimed experts online that share misleading information about food and nutrition that can shape perceptions and influence diet.^6^ People are therefore regularly exposed to conflicting nutrition information from television, online news, and social media^7^ that undermines these recommendations. Prolonged exposure to inconsistent nutrition information over a period of time has been shown to have detrimental effects on consumer beliefs and healthy dietary intentions.^8^ Inconsistent dietary information may also reduce engagement with recommended nutrition behaviours such as fruit and vegetable consumption.^9^

Previous research has assessed the quality of different types of health information but rarely online health information and even less frequently nutrition information online. For example, patient-facing treatment information,^10^ health news in print newspapers,^11^ and patient-orientated websites^12^ have all been evaluated systematically. A tool developed and applied to health information in newspapers by Robinson et al.^11^ has subsequently been used to assess the quality of print newspapers, including nutrition information,^13^ Brexit-related food issues in the UK,^14^ and media reporting on air pollution-related health outcomes.^15^ In addition, alternative quality assessment criteria have been used to assess media coverage of childhood obesity in UAE newspapers^16^ and the veracity of information shared by social media influencers on Instagram.^17^ Blogs, including recipes, have also been considered, including homemade infant milk,^18^ healthy living blogs,^19^ and the nutrient profile of ‘clean eating’ blogs.^20,21^ In response to the increase of blogs as a public-facing source of nutrition information, the construct of blogs written by Dietitians has been considered,^22^ and content analysis of Dietitian- versus non-Dietitian-authored blogs,^23^ but neither study extended to other types of online content. Studies assessing the quality of online nutrition information have used either newly developed, unvalidated assessment criteria or criteria designed to assess patient-facing health information, which do not transfer effectively to nutrition information and make quality comparison across studies challenging. The DISCERN tool^10^ has been widely used to assess the quality of nutrition-related information online. However, while it is validated with high interrater reliability, it is designed to be patient facing and to assess clinical information such as treatment options, risks/benefits of treatment, and quality of life. These assessment criteria are not suitable for public health nutrition information in a non-clinical setting.

Existing reviews have evaluated existing quality assessment tools for assessing the quality of general online health information, as opposed to nutrition specifically,^24^ and educational blogs aimed at healthcare professionals.^25^ Paterson et al.^25^ identified 151 quality indicators in the literature, noting that most scoring systems lacked evidence of validity. Consistent with prior reviews,^26^ Zhang et al.^24^ concluded that quality was defined and measured differently with different studies using different criteria. Both reviews found that the quality of information varied across websites and concluded that overall quality was problematic. To our knowledge, the existing quality assessment tools currently used to assess the quality of online nutrition information are based on medical guidelines, and although these instruments were designed as a guide for consumers to evaluate health information quality, the number of criteria included makes them impractical to use and questions have been raised about their validity.^24^ A more recent systematic review of the suitability of existing criteria and instruments used in evaluating health information on social media highlighted the need for future research to identify specific assessment tools and quality evaluation criteria for information shared on social media.^27^

Disseminating news through social media has become an integral part of online news distribution and consumption, with users contributing as both content creators and content distributors.^28^ However, social media has been criticised for having ‘disturbed media power structures’, that is, a structure that enables reduced influence of professional media and allows public actors to play a greater role in shaping debate.^29^ Online communication can amplify political misinformation^30^ and encourage unconstructive discussion.^31^ Therefore, in the context of widespread sharing of misinformation and disinformation, it is important to understand the quality of the information that has the potential to be widely shared.^32^

Among social media and microblogging platforms, Twitter is a popular social network for discussing news and nutrition-related information globally.^33^ One crucial function of Twitter is as a platform for information sharing, including Uniform Resource Locators (URLs)^34^ to external online content. The act of sharing content on Twitter is active and demonstrates engagement with the content as research shows that not all online content generates active participation.^35^ On Twitter, information sharing is considered either ‘first-degree sharing’, that is, generating original content and/or posting from an external source, or ‘second-degree sharing’, that is, retweeting a tweet.^36^ Mixed-methods frameworks have been used to carry out qualitative analysis in other areas, such as on climate change commentary on Twitter,^34^ and have used content analysis to assess the emotion of tweets.^36,37^ While these frameworks considered thematic analysis of the narrative, they did not look at the quality of the information shared.

In summary, there is a lack of suitable standardised quality assessment criteria to assess the quality of online nutrition information.^25,27^ Where quality assessment tools have been developed for online nutrition-related information, they have been more specifically focused towards clinical information^10^ or have assumed use by informed readers with existing knowledge.^38^ In addition, source credibility is a key consideration online as this can impact the virality of content.^39^ Therefore, assessment of the media source type, as well as the content type, is essential to understand where high- and low-quality information is being published online.

### Aims

Based on the diverse and often unvalidated quality assessment criteria used in the literature and the lack of a universally accepted tool for assessing online nutrition information, this study aimed to:

- develop a novel tool for objective assessment of the quality of online nutrition information;
- validate the novel assessment tool;
- assess the novel assessment for interrater reliability and face validity;
- pilot test to assess the quality of a sample of online nutrition information published on Twitter and assess the relationship between the source and the quality.

## Methods

The online quality assessment tool (OQAT) was developed and validated in six distinct stages and then used to assess the quality of articles from one 24-h period. First, a literature search was carried out searching for validated tools designed to assess the quality of online information. Second, a framework and criteria were developed based on the literature mapping the quality assessment criteria on the framework. Third, the criteria were discussed and agreed within the research team. Fourth, the quality assessment criteria were piloted on a subset of data and the wording of the criteria was refined and criteria were removed if deemed to be duplication. In the fifth stage, the established criteria were validated against an existing print media assessment tool, and reliability was tested. Finally, upon completion of the validation and reliability testing, the validated OQAT was used to assess the quality of articles from a 24-h collection period, 19 April 2021.

### Development of a novel OQAT

A literature search was conducted to identify articles using tools to assess the quality of online and print nutrition and health information on the Web of Science, PubMed, and the Association for Computing Machinery (ACM) Digital Library in 2020 and updated in 2021. The literature search focused on validated and un-validated tools assessing quality of nutrition information or health information more generally, online and in print news. Papers were excluded if they were not health or nutrition related, they assessed nutrition or health literacy, or they assessed videos, images, or audios such as podcasts.

To develop section one of the OQAT, a framework of quality evaluation criteria, and corresponding indicators, critical to the assessment of online information was constructed, informed by previously evaluated quality assessment tools.^24^ The framework was based on two key research papers from the literature: the validated Robinson tool,^11,13^ which the authors were already familiar with and had previously been used to assess nutrition information in print news, and a systematic review,^24^ which categorised the criteria and indicators used in 165 research studies to assess the quality of online health information for consumers. The Robinson tool was selected as it has been used widely to assess nutrition-specific information in newspapers^13^ and includes objective questions that do not assume the rater has a large amount of prior knowledge of nutrition. The tool also assesses public-facing information rather than information for clinical patients, or practitioners, which differs from other tools, and places emphasis on evidence-based reporting. These were deemed to be important criteria for creating an informative novel tool to assess online nutrition information. The systematic review was selected as it is a comprehensive review of existing validated and unvalidated assessment criteria used to assess health information online.

Five of the 21 questions on the Robinson tool were selected as they were relevant for online information (full details are documented in Supplemental Material S1). Three of which were not explicitly stated by Zhang et al. Misleading news and headlines and causal inference^40^ can have detrimental effects on public health, and therefore, the authors felt it was important to include these questions from the Robinson tool. Two major categories were identified by Zhang et al., content and design. This study only considers content; therefore, all criteria categorised as design were discounted at the first stage. Content-related measures were further classified into five criteria with 28 corresponding indications. The criteria and indicators were selected for the OQAT based on relevance to an individual article as opposed to the wider site, and those being represented in at least 50% of the articles reviewed to ensure robust indicators were selected. Initially, 13 indicators were selected.

Once the framework, criteria, and indicators were defined, the quality indicators were initially piloted on 20 randomly selected URLs from the 24-h collection period, 19 April 2021, and assessed by two trained raters. These represented news articles and blogs. Both raters had formal nutrition education. The criteria were refined based on discussion within the team on the relevance of the criteria and the existence of a high correlation between indicators. The resulting 10 quality assessment indicators were grouped into the three relevant categories as per Zhang et al.: currency, credibility, and reliability, as described below.

1. *Currency* refers to whether the content is up to date. The main indicators include the publication date and when the article was last updated.
2. *Credibility* criteria consider authoritativeness and trustworthiness. Authoritativeness refers to whether the content was contributed by creditable sources and cites credible sources. Trustworthiness is whether a source is truthful or biased. The credibility indicators, as identified by Zhang et al.,^24^ overlap with the technical criteria identified by Eysenbach et al.^26^
3. *Reliability* refers to whether the content of a webpage is understandable for general consumers without a nutrition or science background. It does not consider readability and accessibility of the whole website.

Given the infinite nature of online content, the inclusion of currency was deemed necessary to evaluate whether the article includes up-to-date scientific evidence and policy information. Positive responses to all 10 indicators were considered essential for a high-quality source of nutrition information. Articles were scored positively if they met the criteria and zero if they did not. There were no negative scores. From a minimum of zero, the maximum score achievable was 10. A full breakdown of the marking criteria and instructions can be found in Supplemental Material S2.

After scoring, articles were categorised as poor, satisfactory, and high quality based on the quality score: 0–2 indicated poor quality, 3–6 indicated satisfactory quality, and 7–10 indicated high quality. The three cut-offs were selected based on Rasch analysis (see ‘Statistical analysis’ section), which identified the minimum requirements for each category.

To enable the content analysis and comparison of content type, section two of the OQAT was developed to capture the type of information shared (i.e. news article, blog, press release, video, social media, and promotional) and the original source. This differs from other research evaluating social media content, which has focused on the social media user^41^ and network analysis.^42^ In the context of validating the OQAT, the source and content type were determined by manually evaluating the webpage, with two trained raters, both with formal nutrition training, independently reviewing a subset of URLs and meeting to discuss discrepancies. Content was categorised as per section two of the OQAT, with each article reviewed assigned to a category for *media source type*, that is, *professional blog*, *news article*, or *non-governmental organisation (NGO)*, and a category for *content type*, that is, *blog*, *news*, *advertising*, or *video* (Supplemental Material S1).

### Expert panel evaluation

Face validity indicates whether the criteria measure what the developers intended them to measure.^43^ This was assessed by two independent experts as per the literature.^43–45^ The panel was selected based on their publication record in the area of online information quality, the quality of media reporting, or the role of social media in information literacy. The panel reviewed the assessment criteria and related instructions and provided comments on the clarity and content.

### Validation of the novel OQAT

The novel OQAT was validated against an existing tool developed to assess quality of health information in the print idea.^11^ It was not possible to validate with a high-quality existing tool to assess online nutrition information as this does not currently exist. Therefore, only news articles were used for validation against the validated tool developed to assess UK print news using URLs shared on two randomly selected dates in 2021, 19 April and 12 June. Two trained researchers independently assessed all nutrition-related news articles excluding information not categorised as news (such as blogs). Any significant discrepancies were discussed, and consensus was reached.

### Inter-rater reliability

Inter-rater reliability was carried out to ensure the measure was independent of the raters and could therefore be repeated with different raters. To test reliability, two trained raters, both with formal nutrition training, used the OQAT independently to score a randomly selected subset of the URLs shared on 19 April 2021. A minimum of 50 observations is recommended for reliability testing.^46^ Therefore, this was the minimum number included by authors. This included a random subset of blogs (due to the large number), all news articles, and all press releases. Any significant discrepancies between the two raters were identified, the articles were discussed, and consensus was reached.

### Pilot data collection

Twitter was used to collect URLs^34^ for analysis. Tweet Archiver^47^ was used to automatically webscrape Twitter for posts containing the term ‘nutrition’ or #nutrition during a randomly selected 24-h period in 2021, 19 April 2021, using Random.org. The data were then screened, and those without a URL were discounted. The URLs were then screened for eligibility and relevance, discounting advertising, recipes, original research papers, and articles, which did not relate to human health.

Two trained raters, both with formal nutrition training, used the tool independently to score the relevant URLs identified during the 24-h period. Any discrepancies were discussed, and consensus was reached. Articles were excluded if topically irrelevant, were linked to social media, or were advertising or product promotion. Articles on climate change, animal nutrition, food, and agricultural policy were discounted if they did not directly relate to nutrition and human health. In addition, URLs were discounted if they were part of discussion forums or were in video format.

### Statistical analysis

The Statistical Package for the Social Sciences (SPSS) version 28.0 was used for statistical analysis. The internal consistency of the OQAT quality score was calculated using Cronbach’s alpha. This indicates the degree to which items measuring the same general construct produce similar scores. Validity of quality scores for news articles was determined by the intraclass correlation coefficient. This statistic allows for the calculation of the agreement between the OQAT and the Robinson tool.

As the data were ordinal, a weighted Kappa coefficient^48^ was used to measure the agreement between the two raters. Kappa was chosen to assess the interrater agreement rather than correlation coefficients as correlation coefficients only assess covariation in data and do not reflect agreement on the actual scores. Cohen's Kappa can range from −1 to +1, where 0 represents the amount of agreement that can be expected from random chance, and 1 represents perfect agreement between the raters. Scores > 0.81 can be considered a near-perfect agreement.

For the quality analysis, the Shapiro–Wilk's test was used to test for normal distribution, and a *p*-value of <0.01 was taken for significance. The non-parametric Kruskal–Wallis *H* test was used to compare the OQAT scores of blogs, news articles, and press releases due to the non-normality of the data. Rasch analysis was used for the estimation of cut-off levels that represented distinct levels of the quality of information, using Winsteps.^49^ The dichotomous Rasch model was fitted to two sets of data, one for Rater 1 and another for Rater 2, using the Joint Maximum Likelihood Estimation (JMLE) method, and model assumptions were checked for unidimensionality using the principal component analysis of Rasch residuals (PCAR), and other fit statistics were used to evaluate item fit.^50^ Cut-off points were estimated using a score-to-measure table that compared the relationship between each raw score, from 0 to 10, with Rasch model estimates of the location (JMLE measures) and their standard errors (SE) according to the procedure suggested by Wright.^51^

## Results

### Development of the OQAT

Based on a review of quality assessment tools in the literature designed to assess online health information and print news, the following elements were defined for section one:

*Three criteria*: rules by which the quality of information is judged. Criteria reflect the values held by the evaluator regarding what is important for determining the quality of nutrition information.
*Tenindicators*: observable attributes associated with the webpage content, which indicate whether the webpage content meets a given criterion.

Initially, 14 indicators were identified. However, during the development phase, the criteria were refined based on discussion within the team on the relevance of the criteria and the existence of a high correlation between indicators. The highly correlated indicators, such as publication date, date of last review, and date of next review, were removed, as was citing an author and citing a journal, as this could be determined by asking if the article contained links to high-quality sources.

The final version of section one of the novel OQAT consisted of 10 key quality indicators adapted from Robinson et al.^11^ and additional sources. To ensure suitability for online use, the three criteria were based on the quality assessment from Zhang et al.^24^ (Table 1). All indicators were scored positively, and an article could score between the values of 0 and 10.

**Table 1.** Quality assessment criteria for the novel online quality assessment tool (OQAT). Section one: quality assessment.

Criteria	Indicators
Currency (whether the content is up to date)	Publication date or date of last update
Credibility (authoritativeness and trustworthiness)	Authorship – author name Authorship – credentials Attribution – high-quality peer-reviewed references Attribution – quote a specialist Disclosure – financial or professional disclosures, bias disclosure
Reliability	Adequate and accurate background Headline – true reflection of the article and evidence Does not generalise – from animal or lab studies Does not have the potential to cause undue harm or optimism

Section two of the OQAT is a content analysis codebook (Table 2). URLs were manually reviewed and categorised by media source type and content type to allow for quality comparison by content type. Instructions on categorising URLs are available in Supplemental Material S1.

**Table 2.** Content analysis codebook. Section two: media source type and content type.

**Media source type**
1. Blog – personal
2. Blog – professional
3. Company (products and services)
4. Government organisation (e.g. PHE, FDA)
5. Magazine
6. Non-governmental organisation (NGO)
7. Professional news (e.g. CNN, The Guardian, The Huffington Post, BBC)
8. Research institute/university
9. Social media (e.g. YouTube, Instagram, etc.)
10. Unrelated
**Content type**
1. News article
2. Blog
3. Scientific report – out of scope for analysis
4. Press release
5. Video – out of scope for analysis
6. Picture – out of scope for analysis
7. Social media (e.g. Twitter/Facebook status) – out of scope for analysis
8. Promotional – out of scope for analysis

As part of the OQAT development, the criteria were sent to two independent experts for review. Suggestions were made that the OQAT could be improved by scoring poorly if the article is more than 5 years old. The authors agree with this suggestion given the infinite nature of online content and initially included the ‘date of last update’ as a quality indicator; however, this was highly correlated with publication date and therefore was removed. The inclusion of a publication date allows readers to make an informed decision on the relevance of the evidence. However, highlighting that an article is out of date or updating the content regularly is made as a recommendation in the discussion. Additionally, the reviewers suggested that it would be valuable to better identify articles with multiple links to multiple content, both high quality and low quality. Again, this is discussed in the discussion as part of the recommendations.

### Validation of the OQAT

**Figure 1.**
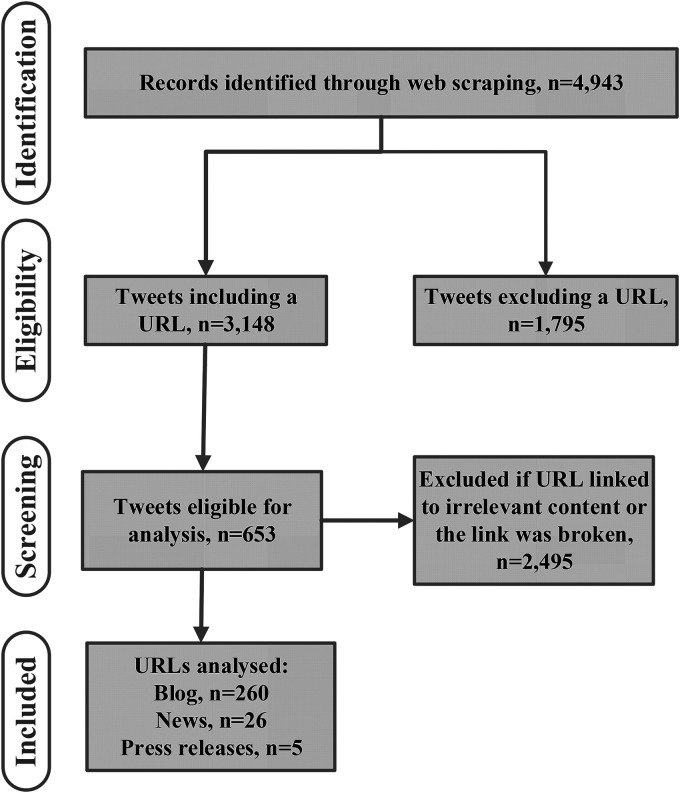
Flow diagram of identification and screening of tweets from 19 April 2021 for analysis to test the novel tool for assessing the quality of online nutrition information.

Following comments from the expert panel, the instructions were refined for clarity and to avoid ambiguity. The panel did not suggest modifying the criteria or indicators; therefore, these were accepted as having face validity. The OQAT was validated against an existing validated tool designed to measure the quality of health information in UK print newspapers^11^ using data scraped from Twitter on 19 April 2021 and 12 June 2021 (due to the limited number of news articles shared on 19 April 2021). Over this randomly selected 24-h period, 2894 tweets were collected from Twitter posts that contained the word ‘nutrition’ or #nutrition, and 1007 posts included a URL. Each URL was reviewed manually (Figure 1) and categorised as per the OQAT codebook (Table 2).

To validate the OQAT, only news articles were assessed as this type of information is what the previously validated tool was designed to assess. A total of *n* = 26 news articles were assessed by two trained raters independently using both the OQAT and the existing Robinson tool.^11^ Raters met to discuss and agree on any discrepancies. Comparison of scores (Table 3) using Cronbach's alpha found moderate internal consistency (*α* = 0.502).

**Table 3.** Tabulation of scores on the Robinson tool and the online quality assessment tool (OQAT) by tertile for news articles.

		OQAT			Total
		Poor	Average	High	
**Robinson tool**	**Poor**	0	2	3	5
	**Average**	0	6	9	15
	**High**	0	0	6	6
**Total**		0	8	18	26

### Reliability testing

After reviewing manually, 291 posts with URLs remained. These represented a total of 260 blogs, 26 news, and 5 press releases. News articles and press releases were oversampled, and 100% of these sources were included due to the small numbers in these categories. Out of the 260 blogs, 100 blogs were randomly selected using Excel’s random generator. Six blogs were excluded as the URLs were broken at the time of review. Therefore, in the final sample to assess interrater reliability, 94 blogs, 26 news articles, and 5 press releases shared on 19 April 2021 (total *n* = 125) were assessed. Discrepancies were identified and discussed, and consensus was reached (Table 4). The weighted Kappa coefficient demonstrated high interrater agreement (*k* = 0.653, *p* < 0.001, 95% CI 0.524–0.782).

**Table 4.** Interrater reliability by tertile for all data assessed.

Before		Rater 2			Total
		Poor	Average	High	
**Rater 1**	**Poor**	2	1	1	4
	**Average**	1	45	10	56
	**High**	0	10	55	65
**Total**		3	56	66	125

### Fit

The Rasch analysis of the data from Rater 1 indicated that 9 out of the 10 items complied with the recommended OUTFIT mean squares between 0.5 and 1.5 for being ‘productive for measurement’ (Table 5). Item 9 had a very low OUTFIT mean square below 0.5 and is ‘less productive for measurement but not degrading’, that is, it does not affect the general fit of the items to the Rasch model. There was a slight difference in the item fits for Rater 2, with most of the same items fitted in the same range, except for item 6 that was in the range of 1.5–2.0 and considered to be ‘unproductive for measurement but not degrading’. All items within these three OUTFIT ranges were acceptable, and no further attempt was made to improve the fit of items. The Rasch model assumption of a single measure that represents a single dimension was confirmed with separate PCAR. The data from Rater 1 had an unexplained variance in the first contrast of 1.83, and the data from Rater 2 had a value of 1.93, both of which was smaller than 2.0 that has been used to indicate likelihood of whether the Rasch measure was unidimensional.^49^ An additional check of unidimensionality examined the values for disattenuated correlation from the comparisons between sets of items that were classified after PCAR. All values were the recommended value of 0.87, indicating that the measure was an adequate measurement of a single latent variable.^50^

**Table 5.** Item fit statistics for the dichotomous Rasch model.

Item^a^	JMLE measures ± SE^b^		OUTFIT mean square	
	Rater 1	Rater 2	Rater 1	Rater 2
Disclosure – 6. Does the article disclose any financial or professional conflict?	8.33 ± 0.28	8.35 ± 0.24	1.24	1.94
Specialist – 5. Does the article quote a specialist?	7.44 ± 0.23	7.61 ± 0.21	1.29	1.34
Reference – 4. Does the article include references to high-quality peer-reviewed resources that can be accessed in one click?	6.73 ± 0.22	6.82 ± 0.20	0.99	0.78
Credentials – 3. Does the article state the authors’ credentials or provide access to a biography?	5.74 ± 0.22	6.74 ± 0.20	0.68	0.71
Result – 10. The article does NOT have the potential to cause undue harm or optimism.	5.07 ± 0.23	5.02 ± 0.23	0.79	0.86
Author – 2. Does the article state the authors’ name?	4.33 ± 0.25	5.24 ± 0.22	0.79	0.78
Accurate – 7. Does the article provide adequate and accurate background?	3.75 ± 0.29	4.85 ± 0.24	0.92	0.74
Representative – 8. Is the headline a true reflection of the article and evidence?	3.67 ± 0.29	4.21 ± 0.28	1.14	1.44
Date – 1. Does the article state the publication date or date of last update?	3.58 ± 0.30	4.12 ± 0.29	0.78	0.79
Generalise – 9. The article does NOT make generalisations from animal or lab studies?	1.4 ± 0.60	0.97 ± 0.98	0.16	0.26

^a^ Question items (indicators) have been ordered based on their order of fit. The estimates for items 10 and 2 were reversed for Rater 2 compared to Rater 1.

^b^ The Joint Maximum Likelihood Estimation (JMLE) estimates have been rescored from their logit values to a measure range of 0–10.

### Estimation of cut-off points

**Figure 2.**
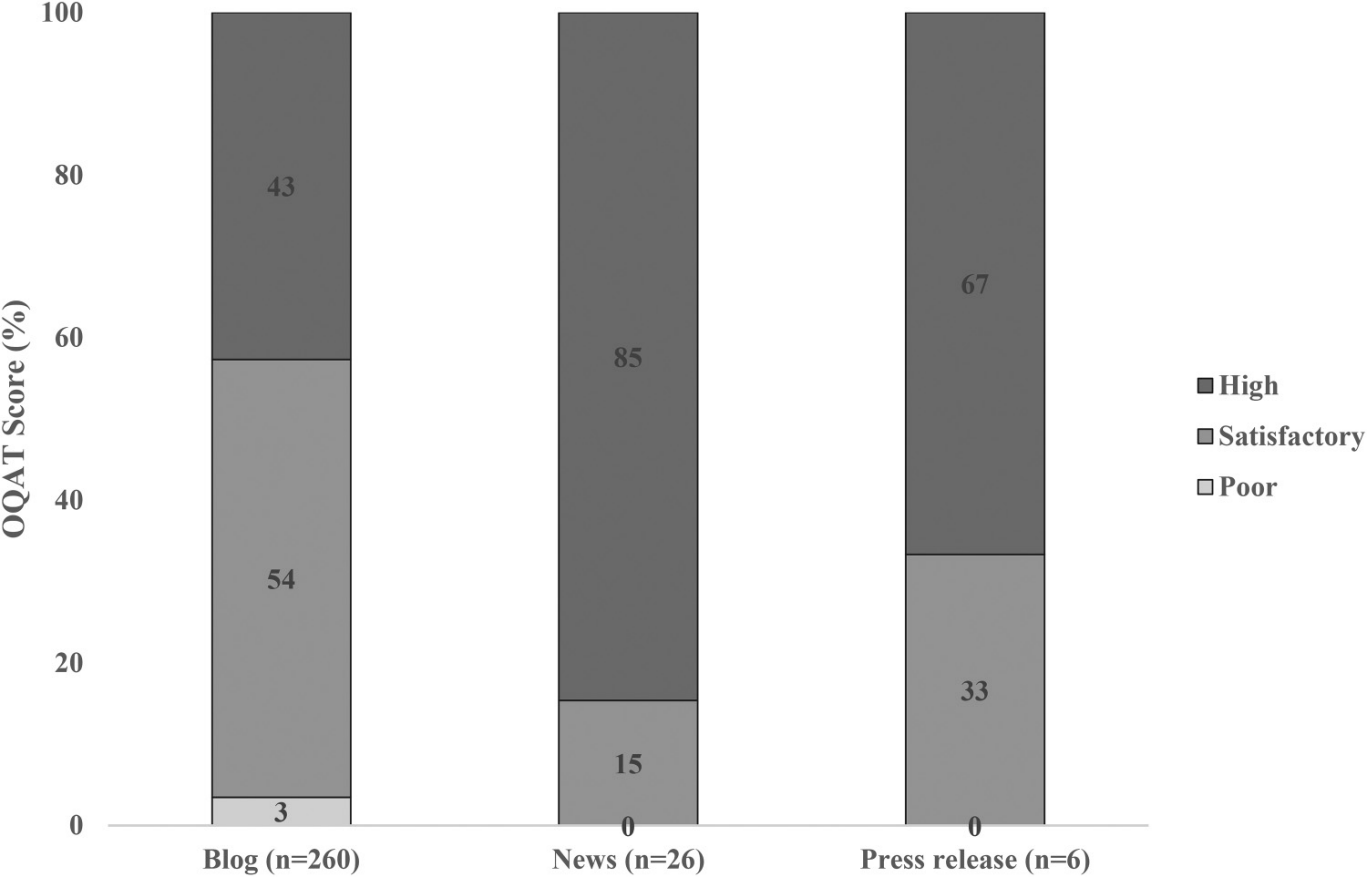
Percentage of articles in each category as assessed by the novel online quality assessment tool (OQAT).

The Wright (person-to-item) Map allows the representation of the questions (ITEMS) and information sources (PERSON) on the single latent Rasch measure. Three statistically independent levels were found for both sets of data independently with the rescaled Rasch cut-off points shown in Supplemental Material as lines in Figures 1 and 2 (Supplemental Material S3). The corresponding raw scores for both sets of data were estimated independently and were low (0–2), medium (3–6), and high (7–10).

### Testing the OQAT for quality assessment of online information

The data scraped from Twitter on 19 April 2021 (*n* = 2894) were reviewed for eligibility (Figure 1) and manually categorised as per the OQAT codebook (Table 2). Articles (*n* = 361) not related to nutrition and human health were excluded. A total of *n* = 291 articles were analysed, and these represented 260 blogs, 5 press releases, and 26 news articles.

The OQAT was pilot tested on the relevant URLs (*n* = 291). The scores generated were not normally distributed, as assessed by the Shapiro–Wilk test (*p* < 0.001). In total, 3% (*n* = 9) of articles were categorised as poor, 49% (*n* = 144) as satisfactory, and 48% (*n* = 139) as high quality (Figure 2).

A Kruskal–Wallis *H* test showed that there was a significant difference in OQAT scores between blogs, news, and press releases, *χ*^2^(2) = 23.22, *p* < 0.001, with a mean rank OQAT score of 138.2 for blogs, 216.6 for news articles, and 188.7 for press releases.

**Figure 3.**
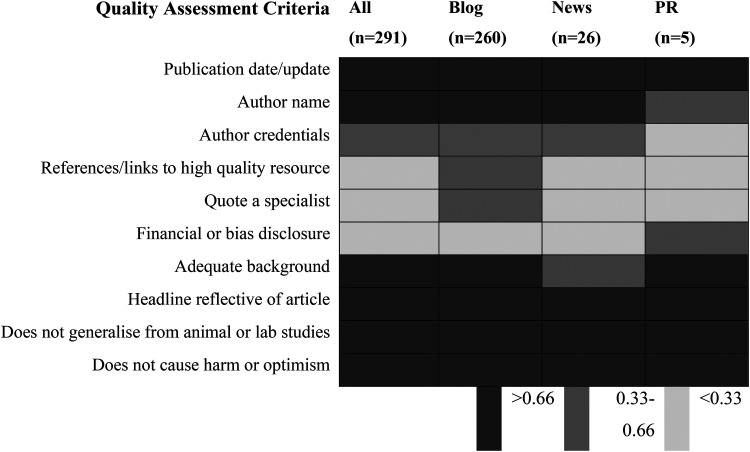
Mean online quality assessment tool (OQAT) score per criterion by content type.

To allow for analysis by criteria, mean scores were calculated for each quality assessment criterion to allow for comparison by criteria and article type. Scores were categorised as positive if they were >0.66 or negative <0.33. Blogs were least likely to state author credentials, quote a specialist, or disclose any financial bias. Across all article types, a specialist quote was least likely to be included as well as disclosure on financial conflict or bias (Figure 3).

## Discussion

Successful development, reliability testing, and validation of this novel OQAT have addressed a gap in the existing literature by providing a validated quality assessment tool suitable for assessing online nutrition information. It is vital that a means of assessing the quality and credibility of online nutrition information is developed due to the lack of current regulation on the internet, the increasingly common access to poor-quality nutrition information, and the growing dependence on online sources for nutrition information. A quality assessment tool is needed to assess changes in the quality of information shared on social media over time if steps are taken in the future to intervene and restrict the spread of nutrition-related misinformation online.

Currently, no such mechanism exists for evaluating nutrition-related online information, although several studies have attempted to assess the quality of a variety of different types of online information. These have included assessment of specific dietary advice for cancer patients,^12^ vegan diets,^17^ and impact of social media influencers.^35^ However, many of these assessment criteria remain unstandardised and/or unvalidated.^24,25^ In addition to the lack of standardisation, most of the tools discussed in the literature were designed and tested before the vast expansion of social media usage and therefore are not well suited to assess the quality of information shared in this way.^27^ Importantly, the initial testing of our novel OQAT instrument, which compared the quality of nutrition information from different sources, demonstrates that our new tool is sufficiently sensitive to detect differences in quality of online information.

An important characteristic of online content is the ability to update the content at any time and its infinite duration. The OQAT was designed to better assess the unique characteristics and wider range of online, freely available public-facing content (which is easy to access, can be written by anyone at any time, and is often discursive yet informative). This differs from other tools designed to assess print news^11,13^ or patient-facing information.^10^ The OQAT captures this key currency information, which is necessarily different to other available tools. The OQAT is also designed to capture the unique online capability of utilising hyperlinks to external sources, such as research journals and author biographies, both of which are key to determining the credibility of the online article and have been shown to impact spread of information.^39^

Validation was found to have moderate internal consistency but perhaps would not be expected to be higher given that online news is provided in a different format than paper-based editions. The validation results show that the OQAT is more suitable for online content than current tools but perhaps would not be suitable for nutrition-related news in print newspapers. It is more inclusive, agile, and more suitable for assessing content that is not based on a traditional press release. Nevertheless, high interrater reliability was reported providing evidence that the assessment tool was robust. The high interrater reliability suggests that the OQAT met the objective to be an objective tool that could be used by trained raters. Face validity was essential to judge the understanding of the criteria and the associated instructions. The expert panel did not recommend any major changes to the criteria; instead, they suggested improving the wording of questions to avoid ambiguity, as seen in other studies in order to improve reliability.^43–45^

Our data show, at least on the day sampled, that blogs were the most prevalent nutrition-related content type shared via Twitter. This has also been found in the context of obesity-related content^52^ but not in the context of online content related to climate change, where news was the most shared^36^ and professional media outlets were the most prolific actors.^53^ This indicates that nutrition articles are different from other topics in the news such as climate change^53^ and politics,^30,54^ which may therefore require a different approach towards the assessment of quality.

An initial assessment of the quality of information indicated that blogs were not only the most prevalent but also the lowest-quality article type. This supports previous work in the literature,^12^ where information in blogs has been measured against dietary advice and found to score poorly on providing scientific evidence and including expert opinion.^12,17,22^ The lack of evidence-based information in blogs found in our study was consistent with the literature pertaining to print news,^13^ obesity,^52^ anti-climate change blogs,^29^ and public-authored political blogs.^31^ All of which identified the damage poor-quality non-expert written blogs can have on public debate. Seeking expert opinion, a sign that the writer was aware of the importance of peer review, was also lacking in many sources, consistent with the published literature.^11^ More encouragingly, the vast majority of articles scored positively on listing an author, an assessment criterion that has previously been shown to positively affect article quality.^11,13^

The main strength of the tool is that it provides a set of standardised assessment criteria, as called for by Afful-Dadzie et al.,^27^ to assess the quality of online content. The quality assessment criteria could expand the OQAT relevance beyond researchers as it could be employed as a checklist by content writers or as a framework for consumers to assess the quality of online nutrition information, providing a motivation for publishing higher-quality information. Similarly, the OQAT may later be suitable for other evidence-based online articles such as more general preventative health information, pending further research.

Further strengths of this study include the OQAT development, which was based on previously validated criteria^11^ and methodology^1,25^ developed and made relevant for online content. However, validation against a tool that was used to inform the development of the OQAT is a limitation. However, there is no gold standard tool to validate against, and there is a lack of validated tools in the literature; therefore, validating the tool using the Robinson tool was deemed the most appropriate method. Data collection was novel in that it used Twitter as the source of URLs, enabling objective selection of a cross-section of content designed to disseminate nutrition information. As Twitter has over 200 million active users, using Twitter ensures that the URLs being assessed have been interacted with. This is preferable to a Google search, which may return content that does not stimulate reader engagement.^35^ By creating a tool that can be used for all nutrition-related online content, the OQAT also builds on recent studies that have previously categorised the positive characteristics of Dietitian-authored blogs^22^ and compared the quality of the blogs to those from lay authors.^55^

This study had several limitations. While a thorough literature review was conducted to identify online and print quality assessment tools and extended to wider health information, some tools may have been missed as a systematic review was not conducted as part of this research. The uniqueness of the OQAT created challenges during the validation process. As the previously validated tool was designed to assess nutrition-related news,^11^ it was necessary to validate the OQAT using only news articles. However, this type of information is not commonly shared on Twitter. This further supports the need for a quality assessment tool that can assess diverse types of online content, as articles categorised as ‘blogs’ are shared more frequently than ‘news’ on Twitter. Similarly, the disproportionately high number of blogs, while representing the type of content being shared, did not allow for a comprehensive comparison of the quality of all content types. A further limitation of the quality assessment methods is that the raters were not blind to the article source. One possible effect of this may have been to moderate the article score if the source was trusted, or not trusted, by the rater. However, the questions were worded as clearly as possible to reduce the risk of bias. A further limitation is that the indicators were not weighted. Rasch analysis indicated that the unweighted items were broadly adequate to assess quality and meet the objective of classifying articles into three quality levels without the need for weighting. However, this is the first iteration of the OQAT and future refined and improved versions may consider weighting.

A limitation of the study, and the OQAT more generally, is that only webpages are considered. Therefore, the wider limitations of a website are not considered, for example, if references cannot be accepted or if author credentials cannot be prominently displayed.

This is because the OQAT was designed to measure online articles as they relate to evidence-based nutrition and not the usability or accessibility of websites, which could include other information such as events or advertising, which were out of scope. Similarly, the OQAT does not include readability scores as these can be assessed by external software such as the Flesch–Kincaid readability test. The OQAT was validated using articles identified with the word and hashtag nutrition and written in English. Relevant information could have been missed if a tweet used alternative descriptive words such as ‘diet’ or ‘healthy lifestyle’. Future research should consider broader search terms. Finally, apart from publication date, the OQAT does not consider how up to date the article is as this is challenging to determine in nutrition as some research and guidelines are relevant 30 years later. Future versions of the OQAT should consider how to reliably deal with this.

Importantly however, the successful development and validation of the OQAT have led to a number of recommendations for practice. Online content and blogs, in particular, are a popular source of nutrition information for the public,^17,22^ but they vary widely in quality.^18,19,52^ Based on the findings from the OQAT development and validation and the wider literature, a series of recommendations to content writers are suggested. Online content that gives dietary advice must be evidence based and provide the evidence to the reader through references or hyperlinks. References and hyperlinks should link to scientific evidence rather than circular links within the website – it is best practice to include an identifying feature for scientifically validated weblinks.

Given the infinite lifespan of online content, articles should be reviewed and updated regularly (annually as a minimum) and include a warning or caveat if the content is more than 5 years old or be removed from the website, so the reader is informed on how up to date the evidence is and not unknowingly exposed to out-of-date nutrition information. Blog authors need to give a brief, referenced summary of the evidence ensuring the most up-to-date evidence is stated to ensure the reader has a comprehensive background of the topic. Additionally, authors should not overstate the evidence. Notably, many blogs reviewed by the OQAT scored poorly as they suggested health can be improved by regularly eating one nutrient or food type over a short period of time, or similar overstatements leading to increased risk of causing undue harm or optimism. Finally, any funding should be explicitly stated so that a reader is informed whether the author has been paid to write about a certain food or topic. Further research is needed to determine whether nutrition information is more likely to be shared if it is of lower quality.

## Conclusions

The development and validation of this novel OQAT add to a body of literature assessing quality of information in the media and online. This study contributes to the methodology of assessing the quality of online information. It has further developed existing tools and guidelines to create a tool that is designed to be simple to use and, with further testing, could be used by non-nutritionists to measure the distinct characteristics of online information. This tool is a reliable and objective method that can be used in future research and practice, either by researchers to assess the quality of online information in different settings or by organisations to inform readers of the quality of information being accessed. While this tool was validated using nutrition information, it may also be suitable for other evidence-based online articles such as more general health information.

## Supplemental Material

sj-docx-1-dhj-10.1177_20552076231187249 - Supplemental material for Development and validation of a quality assessment tool to assess online nutrition information Click here for additional data file. Supplemental material, sj-docx-1-dhj-10.1177_20552076231187249 for Development and validation of a quality assessment tool to assess online nutrition information by Cassandra H Ellis, J Bernadette Moore, Peter Ho and Charlotte EL Evans in DIGITAL HEALTH
