# Supplementary material for: Development and validation of a quality assessment tool to assess online nutrition information
Source: Digit Health. 2023 Jul 16;9:20552076231187249. doi: 10.1177/20552076231187249 (PMC10357061; doi:10.1177/20552076231187249)
Supplement: sj-docx-1-dhj-10.1177_20552076231187249 - Supplemental material for Development and validation of a quality assessment tool to assess online nutrition information [file sj-docx-1-dhj-10.1177_20552076231187249.docx]

**Supplement**

Supplemental material for Development and validation of a quality assessment tool to assess online nutrition information by Cassandra H. Ellis, J Bernadette Moore, Peter Ho and Charlotte E.L. Evans, published in *Digital Health*.

***Supplement 1: Source of the final quality Indicators selected for the novel Online Quality Assessment Tool***

**Criteria Indicator Source**

**Zhang Robinson**

Currency 1. Does the article state the publication date or date 🗸

of last update?

Credibility 2. Does the article state the authors name? 🗸

**3**. Does the article state the authors credentials or provide 🗸

access to a biography?

4. Does the article include references to high quality peer 🗸 🗸

review resources that can be accessed in 1-click?

5. Does the article quote a specialist? 🗸

6. Does the article disclose any financial or professional 🗸

conflict?

Reliability 7. Does the article provide adequate and accurate 🗸 🗸

background?

8. Is the headline a true reflection of the article and evidence? 🗸

9. The article does NOT make generalisations from animal or 🗸

lab studies?

10. The article does NOT have the potential to cause undue 🗸

harm or optimism.

***Supplement 2****:* ***Instructions for using the Online Quality Assessment Tool (OQAT)***

**Introduction**

This Online Quality Assessment Tool (OQAT) has been designed to classify and assess the quality of nutrition news and blogs that have been shared through the microblogging platform, Twitter.

**General instructions**

Three criteria and 10 key indicators will assess the quality of a news articles and blogs. Each of the indicators represents a separate quality criterion. In addition, each article will be categorised by media and content type.

The indicators are organised in three sections as follows:

**Question 1 (currency)** focus on whether the content is up to date, and regularly reviewed and updated.

**Questions 2 - 6** **(credibility)** are concerned with the reliability of the publication and the author, and whether it is a credible source of information.

**Questions 7-10 (reliability)** focus on whether the content can be understood by a lay person. Specifically, does it provide enough background, do any headlines reflect the article, does it make generalisations and does it have the potential to cause undue harm or optimism.

**The rating scale**

Each question is rated on a 2-point scale, *Yes* or *No*. The rating scale has been designed to be a simple yes or no answer. A positive score should be attributed if the criterion is met, a neutral score should be given if the criterion is not met.

Articles can score a maximum of 10, and a minimum of 0. There are no negative scores.

Scores should be categorised as follows:

0-2 – Poor
3-6 - Satisfactory
7-10 – High

**General guidelines are as follows:**

A score of 1 should be given if your answer to the question is a definite 'yes' - the quality criterion has been completely fulfilled. 0 should be given if the answer to the question is a definite 'no' - the quality criterion has not been fulfilled.

After review, each article should be attributed three scores, one quality assessment score, one *Media source type* rating, and one *Content type* rating*.*

**OQAT for nutrition news and blogs**

Section One: Quality assessment

| **Criteria** | **Indicators** | **Score** | |
| --- | --- | --- | --- |
|  |  | **Yes** | **No** |
| Currency | 1. Does the article state the publication date or date of last update? | +1 | 0 |
| Credibility | 2. Does the article state the authors name?  3. Does the article state the authors credentials or provide access to a biography?  4. Does the article include references to high quality peer review resources that can be accessed in 1-click?  5. Does the article quote a specialist?  6. Does the article disclose any financial or professional conflict? | +1  +1  +1  +1  +1 | 0  0  0  0  0 |
| Reliability | 7. Does the article provide adequate and accurate background?  8. Is the headline a true reflection of the article and evidence?  9. The article does NOT make generalisations from animal or lab studies?  10. The article does NOT have the potential to cause undue harm or optimism. | +1  +1  +1  +1 | 0  0  0  0 |

**Classifying content**

In addition to the quality assessment questions, you should also classify the source of the content, and the type of content based on the codebook below. Each article reviewed should be assigned a category, 1-9, for *Media source type*, and a category, 1-8, for *Content type*. Please note that these numbers are categorical.

***Content analysis codebook***

| Section Two: Media source and content type  ***Media source type*** |
| --- |
| 1. Blog – personal |
| 1. Blog – professional |
| 1. Company (products and services) |
| 1. Government organisation (e.g. PHE, FDA) |
| 1. Magazine |
| 1. Non-governmental Organisation (NGO) |
| 1. Professional news (e.g. CNN, The Guardian, The Huffington Post, BBC) |
| 1. Research institute/University |
| 1. Social media (e.g., YouTube, Instagram, etc.) |
| 1. Unrelated |
|  |
| ***Content type*** |
| 1. News article |
| 1. Blog |
| 1. Scientific report - out of scope for analysis |
| 1. Press release |
| 1. Video - out of scope for analysis |
| 1. Picture - out of scope for analysis |
| 1. Social medial (e.g. Twitter/Facebook status) - out of scope for analysis |
| 1. Promotional - out of scope for analysis |

***Supplement 3: Wright Maps used to determine cut-offs for poor, satisfactory and high quality articles***


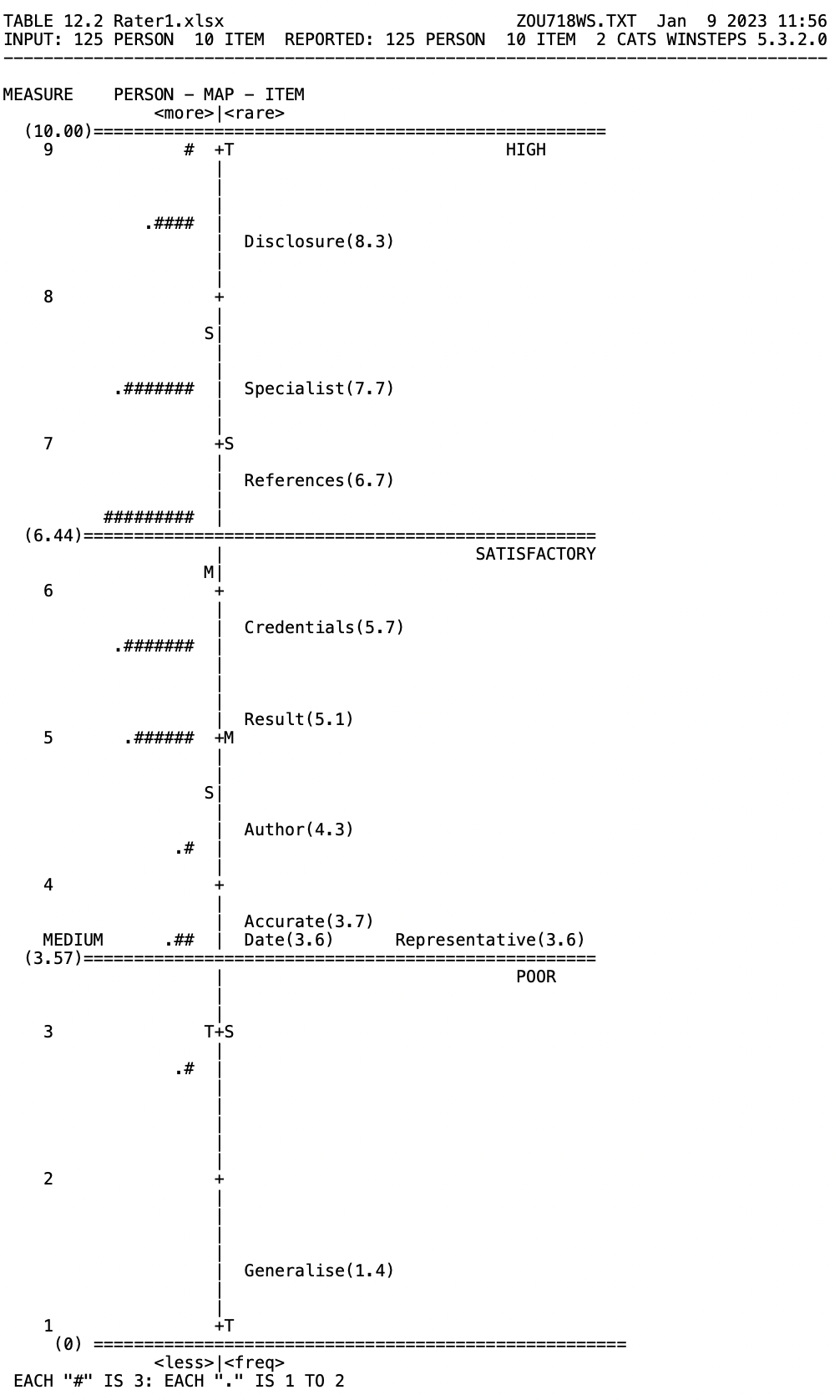


**Figure 1**. Wright Map for Rater 1 of person and item estimates rescaled to raw scores between 0-10 (logits). The symbols “#” and “.” on the left represent 3 and 1 sources (PERSON) and questions (ITEM) are shown on the right with their mean JMLE in brackets. Cut-offs are for HIGH ($\geq$6.44), MEDIUM ($\geq$3.57), LOW ($\geq$0.0). See table 1 for item descriptions.


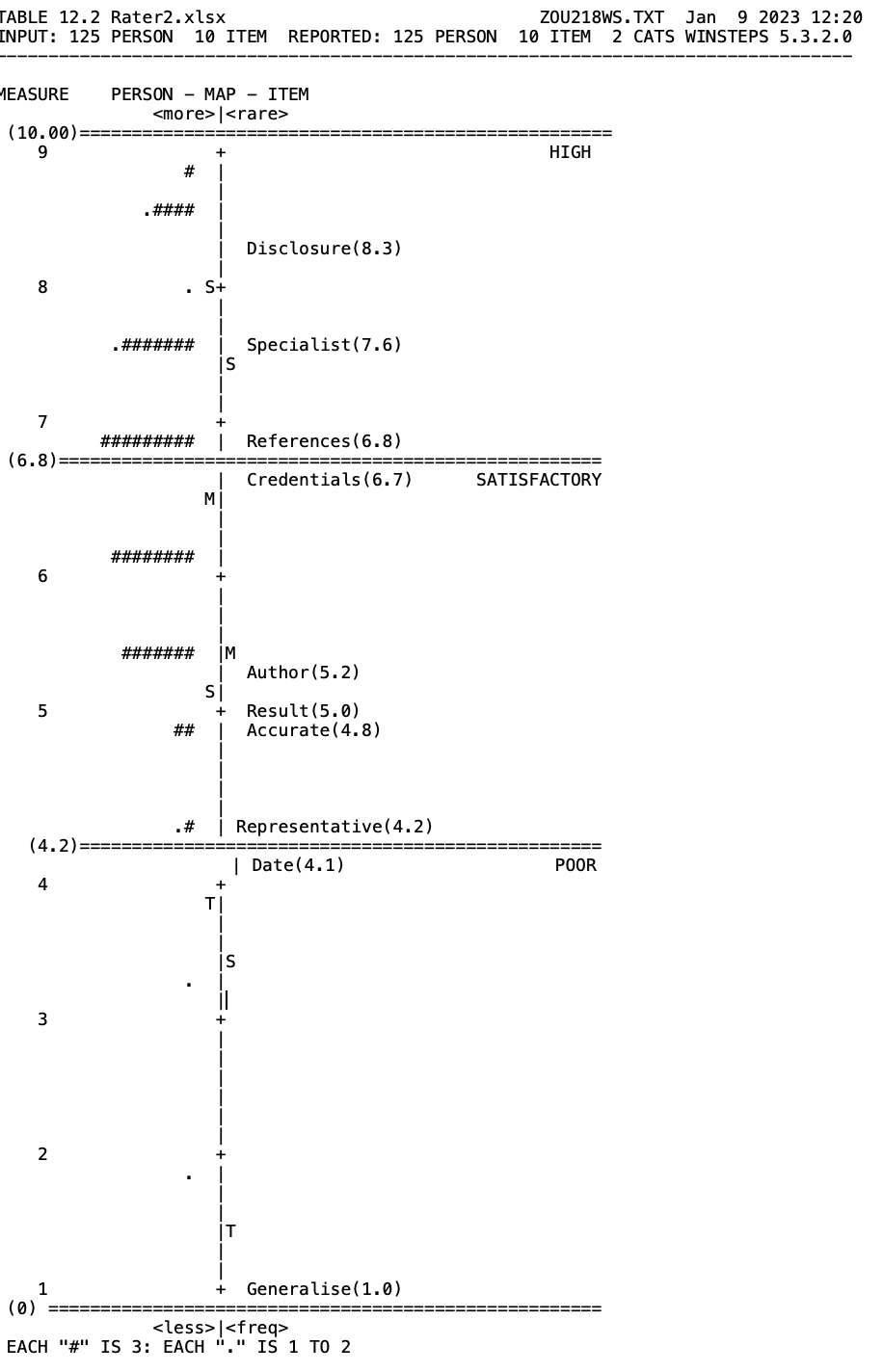


**Figure 2**. Wright Map for Rater 2 of person and item estimates rescaled to raw scores between 0-10 (logits). The symbols “#” and “.” on the left represent 3 and 1 sources (PERSON) and questions (ITEM) are shown on the right with their mean JMLE in brackets. Cut-offs are for HIGH ($\geq$6.8), MEDIUM ($\geq$4.2), LOW ($\geq$0.0). See table 1 for item descriptions.
